# Clinical and Radiographic Outcomes of Immature Teeth Treated with Different Treatment Protocols of Regenerative Endodontic Procedures: A Retrospective Cohort Study

**DOI:** 10.3390/jcm10081600

**Published:** 2021-04-09

**Authors:** Radovan Žižka, Šimon Belák, Jiří Šedý, Kamila Fačevicová, Iva Voborná, David Marinčák

**Affiliations:** 1 Institute of Dentistry and Oral Sciences, Medical Faculty, Palacký University Olomouc, 772 00 Olomouc, Czech Republic; simon.belak@upol.cz (Š.B.); jirisedy@jirisedy.cz (J.Š.); iva.voborna@upol.cz (I.V.); David.marincak01@upol.cz (D.M.); 2 Czech Educational and Dental Research Innovative Group, Vídeňská 6, 639 00 Brno, Czech Republic; 3 Department of Mathematical Analysis and Applications of Mathematics, Faculty of Science, Palacký University Olomouc, Tř. 17. Listopadu 12, 772 00 Olomouc, Czech Republic; kamila.facevicova@gmail.com

**Keywords:** regenerative endodontic procedure, radiographic root area, root development, immature tooth

## Abstract

Regenerative endodontic procedure (REP) is a progressive treatment modality for immature permanent teeth with necrotic pulp. The ambiguousness about the predictability of REP outcome in relation to complete disinfection of the root canal system and the occurrence of discoloration still exists. The aim of this retrospective study was to analyze two treatment protocols on clinical success rate, radiographic root development and the occurrence of discoloration. Eighteen patients were treated by a single operator by either treatment protocol according to the American Association of Endodontists (AAEP, *n* = 9) or a modified protocol (MP, *n* = 9) with the use of 5% sodium hypochlorite and sandblasting. Patients were followed up after 3, 6, 9, 12, 18 and 24 months and clinical success, radiographic root development and the occurrence of discoloration were assessed. The clinical success of MP was significantly higher in two years follow-up (*p* = 0.015), but the change in radiographic root area was higher for AAEP (*p* = 0.017) and the occurrence of discoloration was higher in AAEP (*p* = 0.029). The use of 5% sodium hypochlorite for a longer period of time leads to a higher success rate. The sandblasting of the access cavity reduces the occurrence of discoloration.

## 1. Introduction

Endodontic treatment of immature permanent teeth with necrotic pulp is one of the most challenging treatment options in endodontics because of the thin root wall and open apex. These cases are usually caused by trauma, caries, or a developmental malformation, such as dens evaginatus or dens invaginatus, resulting in pulp necrosis and arrested root development. Regenerative endodontic procedure (REP, also known as revascularization or revitalization) was proposed as an alternative to calcium hydroxide apexification or a calcium silicate apical plug in order to overcome the disadvantageous initial conditions such as a thin root wall or a wide-open apex. In recent systematic reviews, it has been shown that REP has an excellent success rate alone [1] or in comparison to a calcium silicate apical plug [2], but ambiguousness still exists about the predictability of REP outcome [1].

One of the most important factors is the disinfection of the root canal space because it is well understood from the endodontic literature that the elimination of microorganisms from the root canal system is necessary for pulpal and periapical healing [3]. Previous studies have shown that the absence of bacteria is critical for successful revascularization or REP because the new tissue will stop at the level it meets bacteria in the canal space [4,5]. However, the mechanical debridement should be limited because of thin root canal walls, and further weaking could worsen the long-term prognosis. In REP, chemical debridement of the canal system such disinfection irrigants and intracanal medication alone must be relied on. Formerly, it was proposed to use a high concentration of sodium hypochlorite (NaOCl); in most of the studies, higher than 3% concentration of NaOCl has been used [6]. With the development of clinical consideration for regenerative endodontics by the American Association of Endodontists [7] and the European Society of Endodontology position statement of revitalization [8], the use of a low concentration of NaOCl was proposed because the detrimental effect of “full-strength” NaOCl on stem cells of apical papilla or dentin has been shown [9,10]. This proposal manifests in the decline of the use of a high concentration of NaOCl in clinical practice [11,12]. To date, only limited knowledge on the influence of NaOCl concentration on clinical success rate and radiographical root development exists.

Crown discoloration remains an unfavorable complication of otherwise successful regenerative endodontic procedures of immature teeth with necrotic pulp. In recent reviews, the possible mechanisms of discoloration development were discussed [13,14]. To avoid this significant drawback of REP, the use of calcium hydroxide, double antibiotic paste or calcium silicate cement with smaller potential for discoloration was proposed [13,15]. Sandblasting is suggested to use after root canal filling to remove remnants of sealer and other filling materials in order to allow successful and long-term composite restoration [16]. To our knowledge, to this date no study focusing on the effect of sandblasting on the occurrence of discoloration after REP has been published.

Collectively, the objectives of this retrospective study were:

- To evaluate the effect of different irrigation protocols used in REP on the clinical success rate and radiographic root development;
- To analyze the effect of the presence of periapical radiolucency, etiology and time of symptoms’ development after pulp necrosis on the radiographic root development;
- To elucidate the effect of sandblasting on the occurrence of clinical crown discoloration.

## 2. Materials and Methods

### 2.1. Study Population

The study protocol was approved by the Ethical Committee of the Medical Faculty of Palacký University Olomouc, Czech Republic (No. 34/19). We obtained the dental records of all patients who received REPs between years 2014 and 2017 for permanent immature teeth at the Institute of Dentistry and Oral Sciences, Palacký University Olomouc and private practice (Collegium Dentalis, Olomouc, Czech Republic). The treatment was performed by a single operator (R.Ž.).

The inclusion criteria were the following:

- Patients with immature permanent teeth with a diagnosis of pulp necrosis with or without periapical radiolucency who received REP;
- Take part in the follow-up system so clinical and radiographic evaluation was possible if the teeth were asymptomatic.

The exclusion criteria included:

- Immature permanent teeth with a diagnosis of pulp necrosis with or without periapical radiolucency that received a calcium silicate apical plug;
- Immature permanent teeth that received extraction or no treatment.

The population of this cohort study consisted of 18 patients enrolled at the Institute of Dentistry and Oral Sciences, Palacký University Olomouc (*n* = 9) and the Collegium Dentalis, Olomouc (*n* = 9).

### 2.2. Clinical Protocol

All the procedures were performed by a single operator following the recommended protocol by the American Association of Endodontists from February 2013 [7] (AAEP) or by modified protocol (MP). The patients treated with AAEP were treated at the Institute of Dentistry and Oral Sciences, Palacký University, Olomouc and the patients treated with MP were treated in the Collegium Dentalis, Olomouc. At the first appointment, a preoperative periapical radiograph was made, and the teeth were anesthetized with 4% articain with 1:100,000 epinephrine and accessed under rubber dam isolation. Root canals were then disinfected with 20 mL of 1% sodium hypochlorite for 5 min without any activation (AAEP) or 20 mL of 5% sodium hypochlorite for 20 min with passive ultrasonic activation (MP). Passive ultrasonic activation was performed with the U-file ISO 30 (EMS, Nyon, Switzerland) attached to 120° file holder and the working length was shortened 3 mm to working length using the intermittent flush method for approximately 3 min. During the passive ultrasonic irrigation, 5 mL of 5% sodium hypochlorite was used. Calcium hydroxide (Ultracal, Ultradent, South Jordan, UT, USA) was placed as intracanal medicament, and access was sealed with resin composite for 2–4 weeks. At the second appointment, teeth were anesthetized with 3% mepivacaine without a vasoconstrictor in order to not compromise the induction of bleeding, reaccessed, and irrigated with a final rinse with 20 mL of 17% EDTA for approximately 5 min followed by 5 mL of saline. A precurved ISO 25 K-file (VDW, Münich, Germany) was overextended approximately 2 mm apically in order to induce bleeding inside the canal up to the cemento-enamel junction (CEJ). After the formation of a blood clot around 3–4 mm below the CEJ, the calcium silicate cement (ProRoot MTA, Denstsply Sirona, York, PA, USA) coronal barrier was placed. In AAEP, the pulp chamber was cleaned with a wet microbrush, and in MP the cavity was furthermore sandblasted. The sandblasting was performed with Dento-prep (Ronvig, Daugaard, Denmark) and 50 micrometer aluminum oxide particles. The teeth were afterwards adhesively restored with resin composite. A postoperative periapical radiograph was made for all teeth. Patients were scheduled for a follow-up examination after 3, 6, 9, 12, 18 and 24 months.

### 2.3. Data Collection

Data collection from the preoperative visit included patient’s age, sex, tooth type, the recording of clinical signs and symptoms, the response to sensibility tests, the presence or absence of periapical radiolucency, the etiology of pulp necrosis (caries; development anomaly; mild trauma–crown fracture, contusion, subluxation; or severe injury–lateral luxation, extrusive luxation, intrusion or avulsion), and the stage of root development according to Cvek [17]. Data collection at the follow-up visit included the recordings of clinical signs and symptoms, the response to sensibility tests, the presence of tooth discoloration and follow-up periapical radiograph for the assessment of periapical healing and change in root dimensions. The treatment was considered as a clinical success if no symptoms and signs of apical periodontitis were present. Discoloration has been evaluated separately at the last follow-up.

### 2.4. Radiographic Assesment

**Figure 1:**
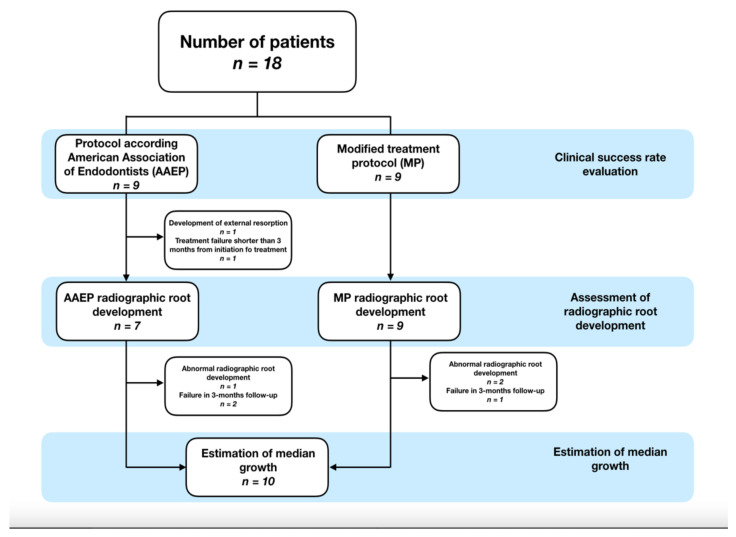
Flow chart of patient exclusions.

Immediate postoperative and follow-up periapical radiographs were first resized to the same size and pixels. Radiographs of each case were then aligned and normalized to each other using the TurboReg plugin tool (Phillipe Thévenaz, Biomedical Imaging Group, Swiss Federal Institute of Technology Lausanne, Lausanne, Switzerland) with the freeware ImageJ (ver. 1.47, National Institute of Health, Bethesda, MD, United States of America). For radiographic analysis, teeth with signs of resorption (*n* = 1) and treatment failure shorter than 3 months from initiation of treatment (*n* = 1) have been excluded (Figure 1). The main examinator Š.B. was not aware of which protocol was used in assessed teeth. 

#### 2.4.1. Periapical Index Measurement

The apical lesions on the radiographic images were quantified and scored by two examiners (Š.B. and R.Ž.) according to the periapical index (PAI) [18]. The examiners were calibrated between themselves on a set of 20 reference radiographs before evaluation. Each apical status was scored from 1 to 5; the PAI score 1 indicated normal periapical structure; PAI 2—small changes in bone structure; PAI 3—changes in bone structure with some mineral loss; PAI 4—periodontitis with well-defined radiolucent area; PAI 5—severe periodontitis with exacerbating features. The included images that were most similar to the reference images were selected, and their scores were recorded. All PAI scores were re-measured by the same examiners one week after the first measurement.

#### 2.4.2. Radiographic Root Length Measurement

The radiographic root length (RRL) was measured according to Bose by a single examiner (Š.B.) who underwent a calibration session with other authors [18]. The ‘‘straight-line’’ tool of TurboReg was used to measure the root length. The root length was measured as a straight line from the CEJ to the radiographic apex of the tooth three times in the first session. A re-measurement was conducted one week after the first measurement.

#### 2.4.3. Radiographic Root Width Measurement

The radiographic root width (RRW) was measured according to Bose et al. by a single examiner (Š.B.) who underwent a calibration session with other authors [19]. The ‘‘straight-line’’ tool of TurboReg was used to measure the root width. The dentinal wall thickness for all radiographs was measured at the level of the apical one third of the preoperative root canal length measured from the CEJ. The root canal width and the pulp space were measured at this level, and the remaining dentin thickness was calculated by subtracting the pulp space from the root canal width. This measurement was made three times in the first session. A re-measurement was conducted one week after the first measurement.

#### 2.4.4. Radiographic Root Area Measurement

The change in radiographic root area (RRA) was calculated as previously described by Flake et al. [20]. The evaluator Š.B. underwent a calibration session with other authors. The measurement was made three times in the first session and a re-measurement was conducted one week after. Despite the fact that the software provides automated measurements, the evaluation was made twice with a one-week time span and the intra-rater agreement was calculated. The RRA measurement was calculated as a percent change between the total root area and the root canal space in each radiograph as follows:

RRA = total root outline–root canal space.

### 2.5. Statistical Analysis

The main analysis focused on the development of RRL, RRW and RRA within time, and dependence on the additional features (type of the protocol, Cvek’s classification, etiology or time of symptoms’ development) was performed with a linear mixed effect model [21]. In order to capture the general features within the data, the variation in the time effect for single patients was treated as random and the remaining effects (of the additional features) were considered fixed.

The subsequent more detailed analyses of differences between groups formed according to treatment protocol, etiology, time of symptoms’ development, clinical success or presence of periapical lesions were performed with the Fisher’s exact test. Due to insufficient counts within the cells of the given table, the *p*-values were obtained by the Monte Carlo simulation.

Since the values of RRL, RRW as well as of RRA were measured three times in the first session by a single observer (Š.B.) and re-measured one week after, the intra-observer reproducibility was verified based on the intraclass correlation coefficients (ICCs) [22]. The ICC was also used in order to verify the interobserver reproducibility of PAI measurements (between R.Ž. and Š.B.).

All the statistical analyses were performed on the level of significance α = 0.05 with the help of the statistical software R and packages lmer4 [23,24], lmerTest and psych [25,26].

## 3. Results

Eighteen patients were assessed during follow-up according to treatment protocol. There was no drop-out, but two teeth were excluded from radiographic analysis because of the development of external replacement resorption and the failure of treatment 2 months before the first check-up (Figure 1). Characteristics such as gender, age, tooth position, and other clinical features are presented in Table 1. Based on the results of the Fisher’s exact test, the value of the Cvek’s classification, etiology or time in the development of symptoms did not depend on the applied protocol (AAEP or MP). The only difference between protocols was observed in discoloration, which tends to be more present by AAEP.

### 3.1. Clinical Success

There was no difference in clinical success (no signs or symptoms of apical periodontitis) in 3, 6 and 24 months follow-up when compared to etiology, time of the development of symptoms and the presence of periapical lesions. None of these factors influence short- or long-term outcome. There was a significant difference only in 24 months follow-up between the AAEP and MP group (*p* = 0.015). The respective odds ratio was estimated as 0.05 and the success rate for teeth treated with MP is therefore significantly higher than for teeth treated with AAEP.

### 3.2. Radiographic Root Length Change

**Figure 2:**
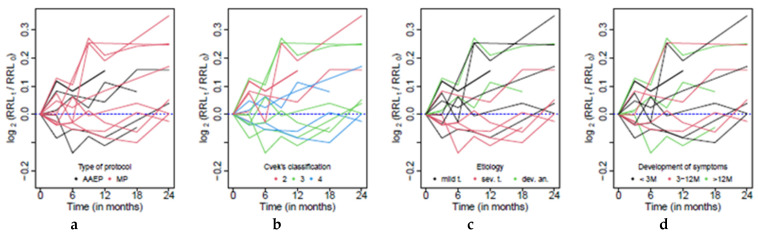
Graphical representation of radiographic root length (RRL) change in time in view of the (**a**) Type of protocol; (**b**) Cvek’s classification; (**c**) Etiology; (**d**) Time of development of symptoms.

The level of intra-observer agreement was considered excellent based on the value of the intraclass correlation coefficients. For the agreement of a set of three measurements in a given time, the ICC was estimated as 0.999 (*p* < 0.001) and the agreement between the first and the second measurements was estimated as 0.998 (*p* < 0.001). Within the further analysis of the development of RRL in time, its relative changes compared to the postoperative value (t = 0) were used. Moreover, these relative changes were symmetrized using logarithm. The analyzed values were therefore given as:

(1) $$\log_{2}\frac{RRL_{t}}{RRL_{0}},\;for\;t=0,\;3,\;6,\;9,\;12,\;18,\;24$$

and its development for each patient is reported in Figure 2.

**Figure 3:**
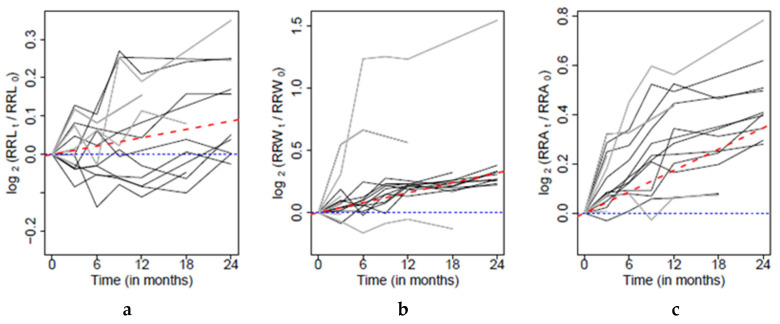
Graphical representation of the median growth (red dashed line) of the radiographic root length (**a**), width (**b**) and RRA (**c**). Data from patients included in the estimation are plotted by black lines, and grey lines represent patients with an anomalous postoperative development.

The overall trend of changes in the radiographic root length (as well as RRW and RRA) was analyzed on the basis of the data from patients who remained in the study longer than 3 months, i.e., measurements from at least the first two controls after the operation were available (four patients excluded because of this reason) and no signs of resorptions (one excluded patient). Moreover, three patients were excluded from this part of the study due to anomalous postoperative radiographic development, which affected the change in the radiographic root width the most. In two of these patients, the endodontic treatment was initiated shortly after injury, and the third patient was excluded because of faulty radiographic alignment. The analysis of the development of RRL, RRW and RRA in time was therefore performed on data from ten patients (Figure 1). The postoperative progression of all patients is visualized in Figure 3.

First of all, the overall trend was estimated. In Figure 3a it is clearly visible that the postoperative development of RRL differed significantly among patients. The resulting model shows that the median growth of the radiographic root length in comparison to its postoperative value equals 0.75 percent every three months (*p* = 0.021). This feature is clearly visible in Figure 3a.

As a next step, the effect of the treatment protocol, Cvek’s classification, time of the development of symptoms and etiology was analyzed in separate mixed effect models. While the effect of time on RRL change did not significantly differ according to the treatment protocol, Cvek’s classification or time of the development of symptoms, there was a significant effect of the etiology. The highest relative growth of RRL appears with patients with a development anomaly, where a median growth by 3% per three months was observed; this effect is moreover comparable to the effect of time by patients with a mild trauma (*p* = 0.170). A significantly lower effect was observed in patients with a severe trauma (*p* = 0.045); within this group the effect of time is rather negligible (*p* = 0.774). These effects are also visible in Figure 2c.

### 3.3. Radiographic Root Width Change

**Figure 4:**
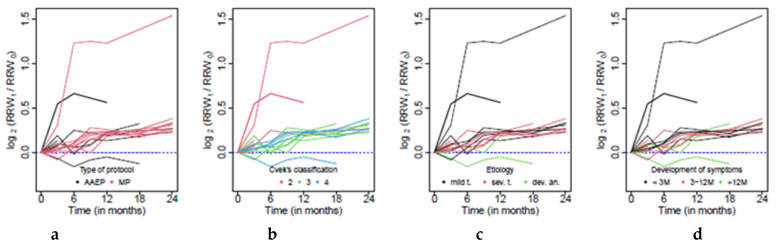
Graphical representation of radiographic root width (RRW) change in time in view of the (**a**) Type of protocol; (**b**) Cvek’s classification; (**c**) Etiology; (**d**) Time of development of symptoms.

Additionally, in the case of the radiographic root width values, the level of intra-observer agreement was examined with an intraclass correlation coefficient, similarly to the case of RRL. In this case the agreement was also considered excellent since the estimated values were 0.998 (*p* < 0.001) and 0.998 (*p* < 0.001) for the agreement of the repetitions and the agreement within time, respectively. The further analysis of the development of RRW in time was performed on its relative changes compared to the postoperative value (t = 0). The relative changes were symmetrized using logarithm. The analyzed values were therefore given as:

(2) $$\log_{2}\frac{RRW_{t}}{RRW_{0}},\;for\;t=0,\;3,\;6,\;9,\;12,\;18,\;24$$

and its development for each patient is reported in Figure 4.

Figure 4 clearly shows the presence of three patients with a nonstandard course of postoperative healing: two with a significantly higher growth of RRW and one for which a decrease in the radiographic root width was observed. These patients were excluded from the analysis of the overall trends of the RRW change in time, as well as those related to changes in RRL and RRA. A more detailed case study of these patients is given in Discussion.

Figure 3b shows a clear increasing trend of RRW change, which is common for all patients. The overall trend was estimated with a mixed effect model, and according to the resulting model the median growth of the radiographic root width in comparison to its postoperative value equals 2.8% every three months (*p* < 0.001). This feature is clearly visible in Figure 3b. The systematic relative growth of the RRW is also observable within groups formed according to the treatment protocol, etiology, time of the development of symptoms and Cvek’s classification, respectively, when it is not significantly affected by the level of any of these factors.

### 3.4. Radiographic Root Area Change

The value of intra-observer agreement was measured with an intraclass correlation coefficient, which in both cases does significantly differ from zero. The estimated values were 0.999 (*p* < 0.001) and 0.999 (*p* < 0.001) for the agreement of the repetitions and the consistency of measurements within time, respectively.

**Figure 5:**
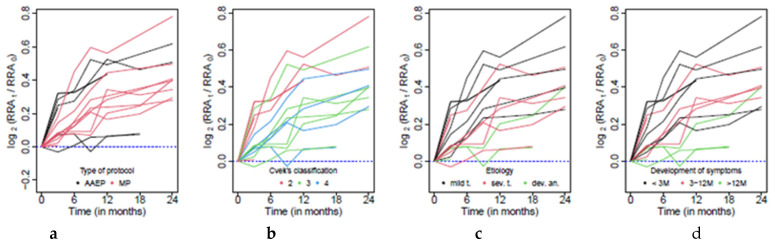
Graphical representation of RRA change in time in view of the (**a**) Type of protocol; (**b**) Cvek’s classification; (**c**) Etiology; (**d**) Time of development of symptoms.

The further analysis of development of RRA in time was performed on its relative changes compared to the postoperative value (t = 0). The relative changes were symmetrized using logarithm. The analyzed values were therefore given as:

(3) $$\log_{2}\frac{RRA_{t}}{RRA_{0}},\;for\;t=0,\;3,\;6,\;9,\;12,\;18,\;24$$

and its development for each patient is reported in Figure 5.

A clear increasing trend is visible in Figure 3c. Therefore, the radiographic root area significantly grows in time, and even though the intensity of the growth varies over patients, the median growth was over all patients estimated at approximately three percent every three months, and after two years it grows by 26.4% (*p* < 0.001). This feature is clearly visible in Figure 3c. Moreover, this trend is comparable for all categories formed by the treatment protocol, etiology, time of the development of symptoms and Cvek’s classification, respectively. On the other hand, a more detailed analysis based on measurements two years after the operation (performed using a standard linear regression model) showed a significant difference between groups formed according to the applied treatment protocol (*p* = 0.017). In patients with the AAEP the median growth after two years was approximately 47.8%, when by the MP it was 29.7%.

### 3.5. Discoloration

The discoloration of the clinical crown was more frequent in the AAEP group (*p* = 0.029).

### 3.6. Periapical Index Change

The intra- and inter-observer agreement was high and the ICC value in both cases does significantly differ from zero. When for the intra-observer agreement the ICC was estimated at 0.931 (*p* < 0.001), for the interobserver agreement it was 0.860 (*p* < 0.001). The distribution of patients according to the value of PAI and selected additional factors observed at the end of the study (after two years) is given in Table 2.

## 4. Discussion

The current retrospective study aimed to analyze the influence of different irrigation protocols and as well other confounding factors on the clinical and radiographic outcome of REP. This could help to identify the complicated cases that are expected to pose some challenges during REP and recognize the limitations of the clinical protocols used to treat these cases. Clinical outcomes of REP are, according to recently published meta-analysis, excellent [1,27,28], even in comparison with the calcium silicate apical plug [2]. However, limitations of the currently published data include a tremendous variability in clinical protocols [11] as well as diverse outcome measurements. The AAE recommends that an ideal REP outcome should include the following three goals: 1) primary goal: the elimination of symptoms and evidence of bone healing; 2) secondary goal: an increase in root development; 3) tertiary goal: a positive response to vitality. The factors influencing the primary and secondary factors might be different. Notably, the different concentrations of sodium hypochlorite, different intracanal medicaments and their concentration are considered to be significant predictors of the clinical success of REP [15], and age [29], the size of the apical foramen [30] and etiology affect the increase in root development [31].

In our study, the clinical success in two-year follow-up was lower for AAEP where 1% sodium hypochlorite was used. Formerly, the concentrations of used NaOCl were generally higher and only in 1% of treated cases the 1% NaOCl was used [6]. Up to the end of 2014, the 1% concentration of NaOCl was used only in a limited number of studies [11]. The decline in the use of higher concentrations of NaOCl is distinguishable in a web-based survey of members of AAE, where in the first visit 60% of participants used 1.6% or higher concentrations of NaOCl, and in the second visit only 31.1% participants used 1.6% or higher concentrations of NaOCl [12]. A current irrigation protocol, suggested by the American Association of Endodontists, follows the results of laboratory studies rather than case reports, which were published previously. The focus of irrigation was redirected from an aggressive disinfection of the root canal system to selective protection of the dentine surface and stem cells, especially in the region of apical papilla [32]. The lower concentration of NaOCl showed a higher risk of failure and was associated with a significantly smaller increase in RRA compared with 6% NaOCl [15]. This could be caused by a huge heterogeneity of this retrospective study because formerly there was higher employment of antibiotic pastes of higher concentrations [6,11,12]. Despite the fact that the high concentrations of NaOCl are detrimental to stem cells [9,10], bacterial elimination and an adequately debrided root canal with a reduction in inflammation might be a prerequisite for optimal reparation [5,15].

Another possible explanation of the low clinical success rate of REP in the AAEP group is the combination of 1% NaOCl and calcium hydroxide as an intracanal medication. In recent laboratory studies, it has been shown that calcium hydroxide provides similar anti-biofilm effects as 500 mg/mL double antibiotic paste [33], but another study showed that triple and double antibiotic pastes were more efficient than calcium hydroxide in the elimination of bacteria from a simulated root canal [34]. Furthermore, dentin treated with antibiotic paste has long residual antimicrobial effect [35]. In clinical studies, there is evidence that the use of triple or double antibiotic had higher success rates than the use of calcium hydroxide [15]. As mentioned before, the proper root canal disinfection is more than relevant for clinical success and subsequent reparation or regeneration. In systematic analysis of failed REP, it has been found that the time elapsed between the initiation of REP and the recognition of failed REP was more than one year in 63% of failed REP cases. Furthermore, 39% of all included failed REP cases were identified at least two years after the initiation of REP [36]. The most common cause of REP failure was a persistent intracanal infection, which was present in 79% of cases [36]. In this study, most of the cases failed in the first year and mostly in the AAEP group where calcium hydroxide and lower concentrations of calcium hydroxide were used. We assume that in this treatment protocol the disinfection of the root canal system did not lead to long-term elimination of bacterial infection from the root canal system.

The secondary AAE goal of an increase in root development is largely achieved in the majority of published REP studies [1,2] and was assessed by a recent systematic review [28]. The meta-analysis showed that 87.4% of the cases exhibited the increment of RRA, and this was found to be higher than the rate of the RRL and RRW increase. Nevertheless, if a 20% radiographic change was used as a clinically meaningful cutoff point, only 34.9% of the samples showed an increment of RRA. The clinical meaningful root development after REP remained unpredictable (16.1% root lengthening and 39.8% root thickening). This study demonstrated a 26.4% difference in RRA between the follow-up and 2-years postoperative time point. This trend was comparable for all categories formed by the treatment protocol, etiology, time of the development of symptoms and Cvek’s classification, respectively. On the other hand, a more detailed analysis based on measurements two years after the treatment (performed using a standard linear regression model) showed a significant difference between the AAEP and MP (*p* = 0.017). The teeth treated with the AAEP had a median RRA change after two years of approximately 47.8%, when with the MP it was 29.7%. We proposed that the use of low concentrated NaOCl could be beneficial for an increase in RRA if the disinfection of the root canal system can be achieved. The criteria for the choice of patients benefiting from the use of a low concentration of NaOCl and maintaining a high success rate should be elucidated in future studies. On the other hand, the change in root length was more eminent in teeth with an etiology of mild trauma or development anomaly. This is in concordance with studies that suggest severe trauma has deleterious effects on the Hertwig’s epithelial root sheath and apical papilla [31,37]. Although we did not assess the apical diameter in our study, a younger patient and more immature teeth have a wider apical foramen, a factor strongly associated with revascularization in the traumatology literature [38]. In clinical studies, it has been shown that the larger size of the apical foramen had positive effects on root thickness, length and root narrowing [39] as well as on clinical success [30]. A further factor which can influence root development after REP was the age of the patient. It has been shown that in patients older than 13 years the increase in RRL is significantly smaller in comparison to younger patients. This effect was not explicit for narrowing apical foramen or root canal wall thickness if a wide apical foramen was present [39]. Other clinical studies have shown a noticeable increase in RRA in more immature teeth [29,37] or RRL [29]. In our study, all patients were younger than 13 years and no statistically significant trend for younger patients was observed.

Another factor which influences the outcome of REP is the etiology of pulp necrosis. There is huge heterogeneity in published studies and only a limited number have evaluated the relative risk between different etiologies. Most of the studies suggest that trauma (and especially severe) has deleterious effects on the apical papilla and Hertwig’s epithelial root sheath [31,37]. This has an impact on the lower success rates of REP and root morphology outcome [40]. On the other hand, some studies suggest that trauma was not risk factor [15]. However, in the last mentioned study, no case after severe trauma such as intrusion or avulsion was treated by REP and the vast majority (73%) of trauma etiology cases were mild in nature (such as fracture or subluxation). In our study, the etiology of pulp necrosis did not have an influence on clinical success. In the graphical representation of RRL change, there were limited signs for the lengthening of severely traumatized teeth. Additionally, the change in RRA for traumatized teeth seemed bigger than for teeth with development anomalies. These findings were not statistically significant. It has been shown that for development anomalies such as dens evaginatus, the REP represents a very suitable treatment modality [30], with higher clinically significant radiographic root development [28]. However, it might be because of the presence of vital pulp tissue in the apical part because this clinical scenario can be treated by deep pulpotomy as well [41]. It has been shown that residual pulp tissue in the apical part of the root canal had a positive effect on REP in in vivo studies [2]. This presence of vital pulp could be likely related to the time of the development of symptoms. The earlier it is after pulp exposure after cusp fracture in dens evaginatus, the probability of pulp persistence will increase, similar to pulp exposure after dental traumatic injury. In the present study, if symptoms developed or REP was initiated in up to 12 months after traumatic dental injury, the change in RRA on graphical representation was higher, although not statistically significant. The initiation of REP in teeth with development anomalies was higher than 12 months because all of the cases with development anomalies were diagnosed as dens invaginatus and two of three cases failed during follow-up. This could be caused by the more complicated internal anatomy of dens invaginatus in comparison to dens evaginatus; therefore, the bacterial elimination from the root canal anatomy could have been less efficient. In a similar way, the carious etiology of pulp necrosis was found to be a risk factor [15].

It was anticipated that the clinical scenarios with more established bacterial infection could have a lower success rate and radiographic root development after REP [42,43]. Nevertheless, according to available studies, the initial diagnosis has a negligible effect on success rates [40,44]. On the other hand, the presence of periapical radiolucency before REP was related to lower radiographic root development on 2D radiographs [15] or 3D scans [45]. Moreover, no periapical radiolucency was completely healed on 3D scans during follow-up [45]. In our study, we observed no influence of diagnosis and presence of periapical radiolucency on clinical success rate or change in PAI.

**Figure 6:**
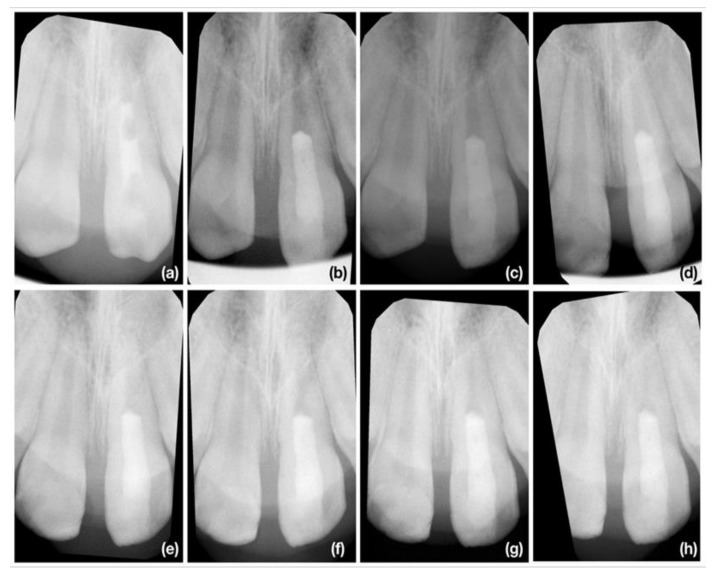
The illustrative case of the most successful courses after regenerative endodontic procedure (REP). The 8-year-old girl was referred for endodontic treatment 2 months after dental traumatic injury of tooth 21 (non-complicated crown fracture). (**a**) The diagnostic X-ray of tooth 11 and 21. There is presence of periapical radiolucency of tooth 21; (**b**) The X-ray taken immediately after finishing REP; (**c**) The control X-ray at 3-months follow up. The periapical lesion of tooth 21 was healed. There are no evident signs of newly produced mineralized tissue; (**d**) The control X-ray at 6-months follow up. There are signs of increase in root canal wall thickness along whole root up to coronal barrier; (**e**) The control X-ray at 9-months follow up; (**f**) The control X-ray at 12-months follow up; (**g**) The control X-ray at 18-months follow up; (**h**) The control X-ray at 24-months follow up. Progressive obliteration of root canal system is evident. There is present radiolucency under coronal barrier.

**Figure 7:**
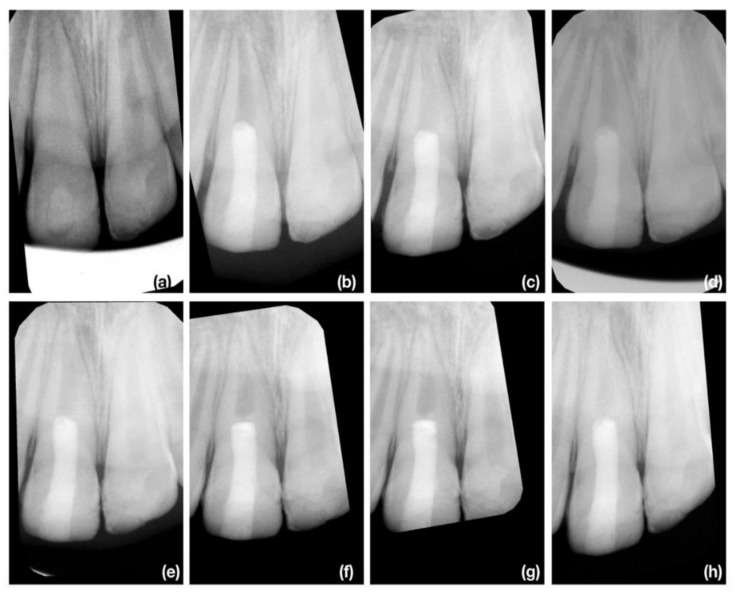
The illustrative case of apical apposition of newly produced mineralized tissue after REP. The 9-year-old boy was referred for endodontic treatment 8 months after dental traumatic injury of tooth 11 (non-complicated crown fracture). (**a**) The diagnostic X-ray of tooth 11 and 21. There is presence of periapical radiolucency of tooth 11; (**b**) The X-ray taken immediately after finishing REP; (**c**) The control X-ray at 3-months follow up. The periapical lesion of tooth 11 was healed; (**d**) The control X-ray at 6-months follow up. No definite signs of any apposition of newly produced mineralized tissue; (**e**) The control X-ray at 9-months follow up. The apposition of newly produced tissue is visible in apical part and in the contact with coronal barrier; (**f**) The control X-ray at 12-months follow up. Radiopaque barrier is more evident in contact with coronal barrier; (**g**) The control X-ray at 18-months follow up. Further apposition of mineralized tissue in apical third of root; (**h**) The control X-ray at 24-months follow up. Progressive production of mineralized tissue in apical third of root is evident and pulp canal obliteration is formed apically.

**Figure 8:**
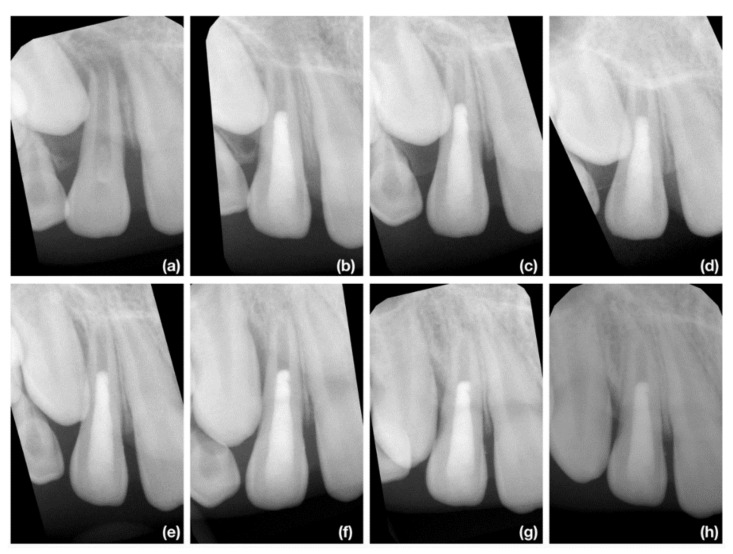
The illustrative case of the least successful course after REP. The 8-year-boy girl was referred for endodontic treatment approximately 12 months after eruption of tooth 12. (**a**) The diagnostic X-ray of tooth 12. The radiopaque invagination is evident in cervical part of tooth; (**b**) The X-ray taken immediately after finishing REP; (**c**) The control X-ray at 3-months follow up. The periapical lesion of tooth 21 was reduced in size. No visible signs of apposition of mineralized tissue; (**d**) The control X-ray at 6-months follow up. There are no signs of apposition of newly produced mineralized tissue; (**e**) The control X-ray at 9-months follow up. In the most apical part of the root mineralized tissue is evident, which fills apical 2 mm of root; (**f**) The control X-ray at 12-months follow up. The amount of produced tissue is similar to 9-months follow-up; (**g**) The control X-ray at 18-months follow up. The newly produced mineralized tissue is thickening with slight apposition on root canal wall in apical third. The mineralization of detached apical papilla is evident; (**h**) The control X-ray at 24-months follow up. Despite some apposition in apical 2–3 mm the radiological appearance is very similar to postoperative X-ray.

We must emphasize that the most successful cases in the recent study were with trauma etiology of pulp necrosis, and symptoms developed to three months after mild dental traumatic injury as illustrated in Figure 6a–h. As these patients were already referred with initiated endodontic treatment, we are not able to exclude the possibility of improper diagnosis state of the pulp after dental traumatic injury. Dissimilar to vital pulp therapy, there was no mineralized tissue in contact with the coronal barrier and the direction of apposition of the newly produced mineralized tissue was from the apical part to coronal. The similar apposition of newly produced mineralized tissue was mostly present in other cases, but was less distinct in cases with an etiology of dental trauma and the initiation of endodontic treatment longer than 3 months (Figure 7a–h) or an etiology of development anomaly (Figure 8a–h)

Importantly, the restoration of pulp sensibility after REP is the tertiary goal of AAE. The regaining of sensibility on thermal tests represents a sign of the presence of sensory innervation and necessary vasculature. In the present study, no positive response to sensibility tests was found in any case, even after two years follow-up. However, the currently available data on this issue are scarce. There is only one study where pulp sensibility was positive to cold and/or the electric pulp test in 50% of REP cases, and these cases were associated with a greater root development than teeth with negative response [15].

Discoloration remains an unfavorable complication of otherwise successful regenerative endodontic procedures on immature teeth with necrotic pulp. There are several possible mechanisms of how discoloration in REP can occur, such as the use of triple antibiotic paste with minocycline, calcium silicate cements with bismuth or blood contamination [13,14]. In the present study, we used calcium hydroxide in both groups to overcome the negative effect of triple antibiotic paste. Despite the fact that we used as a coronal barrier calcium silicate cement containing bismuth (ProRoot MTA), which has a higher potential for discoloration [46], the discoloration was more evident in the AAEP group. It was described that the setting of calcium silicate cement with bismuth in contact with NaOCl leads to discoloration [47,48]. Nevertheless, the use of high concentrations of NaOCl for a longer period of time in the MP group did not increase the occurrence of discoloration. The most probable cause of the lower incidence of discoloration in the MP group is sandblasting the access cavity. This step reduces dentine surface contamination by calcium silicate cement and subsequently reduces the occurrence of discoloration. Another possible solution of calcium silicate discoloration is the use of calcium silicate cement with a lower potential for discoloration [46]. Even then, these materials have the potential for discoloration when they set in contact with irrigation solutions or blood [13]. Especially in REP, we must emphasize that contamination from blood clots can cause discoloration even with the use of calcium silicate cements without bismuth [49]. Sandblasting might be beneficial even when using calcium silicate cement contaminated by blood.

The main limitation of this retrospective study is its relatively small number of patients. We have to point out that a sample size of about 20 patients is the common variation [29,37,50,51,52,53,54,55], with small exceptions such as studies with smaller [55,56,57,58] or larger [15,40] sample sizes. On the other hand, the present study is advantageous over some of the previous studies where, e.g., different operators and heterogeneity in treatment protocols (such as irrigation protocols, intracanal medication) were present. To our knowledge, this is the first time the clinical effect of the concentration of NaOCl on radiographic root development in REP has been studied.

## 5. Conclusions

It seems that the modified irrigation protocols with the use of highly concentrated sodium hypochlorite might have a higher clinical success rate after 2-years follow up and no adverse effects in the mean of radiographic root length or width, but the change in radiographic root area was noticeable when a low concentration of sodium hypochlorite was used. The change in radiographic root length was more eminent if the etiology of pulp necrosis was from a developmental tooth anomaly or mild trauma. The sandblasting of the pulp chamber after the placement of the coronal barrier reduces the risk of crown discoloration. The authors suggest caution with strong conclusions because of the relatively small sample size. Future studies with a larger sample size are needed to confirm the results.

## Figures and Tables

**Table 1:** Description of treatment group epidemiological characteristics. AAEP—protocol according American Association of Endodontists, MP—modified protocol, PAI—periapical index.

	AAEP (*n* = 9)	MP (*n* = 9)	*p*
Age in years, mean	8.44	9.22	-
Male sex	5/9	4/9	-
PAI after 24 M, median	4	3	0.3576
Presence of periapical lesion	7/9	6/9	1
Cvek’s classification			0.522
II	5/9	2/9	
III	3/9	4/9	
IV	1/9	3/9	
Etiology			0.697
Mild trauma	3/9	4/9	
Severe trauma	5/9	3/9	
Development anomaly	1/9	2/9	
Development of symptoms			1
<3 months	4/9	4/9	
3–12 months	3/9	3/9	
>12 months	2/9	2/9	
Discoloration at follow-up	9/9	4/9	0.029
Positive pulp test at follow-up	0/9	0/9	

**Table 2:** Distribution of patients according to values of periapical index (PAI), type of protocol, presence of periapical lesion and etiology observed after two years.

	1	2	3	4	5
Type of protocol					
AAEP	0	1	0	1	1
MP	1	2	4	1	0
Presence of periapical lesion					
Present	1	1	3	1	1
Not present	0	2	1	1	0
Etiology					
Mild trauma	0	2	2	0	1
Severe trauma	0	1	2	2	0
Development anomaly	1	0	0	0	0
Development of symptoms					
<3 months	0	1	3	1	1
3–12 months	0	2	1	1	0
>12 months	1	0	0	0	0

## Data Availability

Not applicable.

## References

[1] Tong H.J., Rajan S., Bhujel N., Kang J., Duggal M., Nazzal H. (2017). Regenerative Endodontic Therapy in the Management of Nonvital Immature Permanent Teeth: A Systematic Review-Outcome Evaluation and Meta-Analysis. J. Endod..

[2] Torabinejad M., Nosrat A., Verma P., Udochukwu O. (2017). Regenerative Endodontic Treatment or Mineral Trioxide Aggregate Apical Plug in Teeth with Necrotic Pulps and Open Apices: A Systematic Review and Meta-Analysis. J. Endod..

[3] Kakehashi S., Stanley H.R., Fitzgerald R.J. (1965). The Effects of Surgical Exposures of Dental Pulps in Germ-Free and Conventional Laboratory Rats. Oral. Surg. Oral. Med. Oral. Pathol..

[4] Yanpiset K., Trope M. (2000). Pulp Revascularization of Replanted Immature Dog Teeth after Different Treatment Methods. Endod. Dent. Traumatol..

[5] Verma P., Nosrat A., Kim J.R., Price J.B., Wang P., Bair E., Xu H.H., Fouad A.F. (2017). Effect of Residual Bacteria on the Outcome of Pulp Regeneration In Vivo. J. Dent. Res..

[6] Diogenes A., Henry M.A., Teixeira F.B., Hargreaves K.M. (2013). An Update on Clinical Regenerative Endodontics. Endod. Top..

[7] American Association of Endodontists (2013). Considerations for a Regenerative Endodontics Procedure. http://www.aae.org/specialty/wp-content/uploads/sites/2/2017/07/considerationsregendo.pdf.

[8] Galler K.M., Krastl G., Simon S., Van Gorp G., Meschi N., Vahedi B., Lambrechts P. (2016). European Society of Endodontology Position Statement: Revitalization Procedures. Int. Endod. J..

[9] Ruparel N.B., Teixeira F.B., Ferraz C.C.R., Diogenes A. (2012). Direct Effect of Intracanal Medicaments on Survival of Stem Cells of the Apical Papilla. J. Endod..

[10] Martin D.E., De Almeida J.F.A., Henry M.A., Khaing Z.Z., Schmidt C.E., Teixeira F.B., Diogenes A. (2014). Concentration-Dependent Effect of Sodium Hypochlorite on Stem Cells of Apical Papilla Survival and Differentiation. J. Endod..

[11] Kontakiotis E.G., Filippatos C.G., Tzanetakis G.N., Agrafioti A. (2015). Regenerative Endodontic Therapy: A Data Analysis of Clinical Protocols. J. Endod..

[12] Lee J.Y., Kersten D.D., Mines P., Beltran T.A. (2018). Regenerative Endodontic Procedures among Endodontists: A Web-Based Survey. J. Endod..

[13] Žižka R., Šedý J., Gregor L., Voborná I. (2018). Discoloration after Regenerative Endodontic Procedures: A Critical Review. Iran. Endod. J..

[14] Kahler B., Rossi-Fedele G. (2016). A Review of Tooth Discoloration after Regenerative Endodontic Therapy. J. Endod..

[15] Chrepa V., Joon R., Austah O., Diogenes A., Hargreaves K.M., Ezeldeen M., Ruparel N.B. (2020). Clinical Outcomes of Immature Teeth Treated with Regenerative Endodontic Procedures-A San Antonio Study. J. Endod..

[16] Schwartz R.S., Fransman R. (2005). Adhesive dentistry and endodontics: Materials, clinical strategies and procedures for restoration of access cavities: A review. J. Endod..

[17] Cvek M. (1992). Prognosis of Luxated Non-Vital Maxillary Incisors Treated with Calcium Hydroxide and Filled with Gutta-Percha. A Retrospective Clinical Study. Endod. Dent. Traimatol..

[18] Orstavik D., Kerekes K., Eriksen H.M. (1986). The Periapical Index: A Scoring System for Radiographic Assessment of Apical Periodontitis. Endod. Dent. Traumatol..

[19] Bose R., Nummikoski P., Hargreaves K. (2009). A Retrospective Evaluation of Radiographic Outcomes in Immature Teeth with Necrotic Root Canal Systems Treated with Regenerative Endodontic Procedures. J. Endod..

[20] Flake N.M., Gibbs J.L., Diogenes A., Hargreaves K.M., Khan A.A. (2014). A Standardized Novel Method to Measure Radiographic Root Changes after Endodontic Therapy in Immature Teeth. J. Endod..

[21] Agresti A. (2015). Foundations of Linear and Generalized Linear Models.

[22] Shrout P.E., Fleiss J.L. (1979). Intraclass Correlations: Uses in Assessing Rater Reliability. Psychol. Bull..

[23] Bates D., Mächler M., Bolker B., Walker S. (2015). Fitting Linear Mixed-Effects Models using lme4. J. Stat. Soft..

[24] R Core Team (2019). R: A Language and Environment for Statistical Computing.

[25] Kuznetsova A., Brockhoff P.B., Christensen R.H.B. (2017). lmerTest Package: Tests in Linear Mixed Effects Models. J. Stat. Soft..

[26] Revelle W. (2020). Psych: Procedures for Psychological, Psychometric and Personality Research.

[27] Koç S., Del Fabbro M. (2020). Does the Etiology of Pulp Necrosis Affect Regenerative Endodontic Treatment Outcomes? A Systematic Review and Meta-Analyses. J. Evid. Based Dent. Pr..

[28] Ong T.K., Lim G.S., Singh M., Fial A. (2020). V Quantitative Assessment of Root Development after Regenerative Endodontic Therapy: A Systematic Review and Meta-Analysis. J. Endod..

[29] Li L., Pan Y., Mei L., Li J. (2017). Clinical and Radiographic Outcomes in Immature Permanent Necrotic Evaginated Teeth Treated with Regenerative Endodontic Procedures. J. Endod..

[30] Fang Y., Wang X., Zhu J., Su C., Yang Y., Meng L. (2018). Influence of Apical Diameter on the Outcome of Regenerative Endodontic Treatment in Teeth with Pulp Necrosis: A Review. J. Endod..

[31] Nazzal H., Duggal M.S. (2017). Regenerative Endodontics: A True Paradigm Shift or a Bandwagon about to Be Derailed?. Eur. Arch. Paeditr. Dent..

[32] Žižka R., Šedý J. (2017). Paradigm Shift from Stem Cells to Cell-Free Regenerative Endodontic Procedures: A Critical Review. Stem Cells Dev..

[33] Tagelsir A., Yassen G.H., Gomez G.F., Gregory R.L. (2016). Effect of Antimicrobials used in Regenerative Endodontic Procedures on 3-Week-Old Enterococcus Faecalis Biofilm. J. Endod..

[34] Latham J., Fong H., Jewett A., Johnson J.D., Paranjpe A. (2016). Disinfection Efficacy of Current Regenerative Endodontic Protocols in Simulated Necrotic Immature Permanent Teeth. J. Endod..

[35] Sabrah A.H.A., Yassen G.H., Spolnik K.J., Hara A.T., Platt J.A., Gregory R.L. (2015). Evaluation of Residual Antibacterial Effect of Human Radicular Dentin Treated with Triple and Double Antibiotic Pastes. J. Endod..

[36] Almutairi W., Yassen G.H., Aminoshariae A., Williams K.A., Mickel A. (2019). Regenerative Endodontics: A Systematic Analysis of the Failed Cases. J. Endod..

[37] Chan E.K.M., Desmeules M., Cielecki M., Dabbagh B., Ferraz Dos Santos B. (2017). Longitudinal Cohort Study of Regenerative Endodontic Treatment for Immature Necrotic Permanent Teeth. J. Endod..

[38] Andreasen J.O., Paulsen H.U., Yu Z., Bayer T., Schwartz O. (1990). A Long-Term Study of 370 Autotransplanted Premolars. Part II Tooth Survival and Pulp Healing Subsequent to Transplantation. Eur. J. Orthod..

[39] Estefan B.S., El Batouty K.M., Nagy M.M., Diogenes A. (2016). Influence of Age and Apical Diameter on the Success of Endodontic Regeneration Procedures. J. Endod..

[40] Lin J., Zeng Q., Wei X., Zhao W., Cui M., Gu J., Lu J., Yang M., Ling J. (2017). Regenerative Endodontics Versus Apexification in Immature Permanent Teeth with Apical Periodontitis: A Prospective Randomized Controlled Study. J. Endod..

[41] Tsukiboshi M., Ricucci D., Siqueira J.F.J. (2017). Mandibular Premolars with Immature Roots and Apical Periodontitis Lesions Treated with Pulpotomy: Report of 3 Cases. J. Endod..

[42] Shetty H., Shetty S., Kakade A., Desai R., Zhang C.F., Neelakantan P. (2018). Cone-Beam Computed Tomographic and Histological Investigation of Regenerative Endodontic Procedure in an Immature Mandibular Second Premolar with Chronic Apical Abscess. J. Investig. Clin. Dent..

[43] Nair P.N.R. (2014). Endodontic Biofilm, Technology and Pulpal Regenerative Therapy: Where Do We Go from Here?. Int. Endod. J..

[44] Linsuwanont P., Sinpitaksakul P., Lertsakchai T. (2017). Evaluation of Root Maturation after Revitalization in Immature Permanent Teeth with Nonvital Pulps by Cone Beam Computed Tomography and Conventional Radiographs. Int. Endod. J..

[45] Shetty H., Shetty S., Kakade A., Mali S., Shetty A., Neelakantan P. (2020). Three-Dimensional Qualitative and Quantitative Analyses of the Effect of Periradicular Lesions on the Outcome of Regenerative Endodontic Procedures: A Prospective Clinical Study. Clin. Oral. Investig..

[46] Możyńska J., Metlerski M., Lipski M., Nowicka A. (2017). Tooth Discoloration Induced by Different Calcium Silicate-Based Cements: A Systematic Review of In Vitro Studies. J. Endod..

[47] Camilleri J. (2014). Color Stability of White Mineral Trioxide Aggregate in Contact with Hypochlorite Solution. J. Endod..

[48] Marciano M.A., Duarte M.A.H., Camilleri J. (2015). Dental Discoloration Caused by Bismuth Oxide in MTA in the Presence of Sodium Hypochlorite. Clin. Oral. Investig..

[49] Chen S.J., Karabucak B., Steffen J.J., Yu Y.-H., Kohli M.R. (2020). Spectrophotometric Analysis of Coronal Tooth Discoloration Induced by Tricalcium Silicate Cements in the Presence of Blood. J. Endod..

[50] Jeeruphan T., Jantarat J., Yanpiset K., Suwannapan L., Khewsawai P., Hargreaves K.M. (2012). Mahidol Study 1: Comparison of Radiographic and Survival Outcomes of Immature Teeth Treated with Either Regenerative Endodontic or Apexification Methods: A Retrospective Study. J. Endod..

[51] Saoud T.M.A., Zaazou A., Nabil A., Moussa S., Lin L.M., Gibbs J.L. (2014). Clinical and Radiographic Outcomes of Traumatized Immature Permanent Necrotic Teeth after Revascularization/Revitalization Therapy. J. Endod..

[52] Alobaid A.S., Cortes L.M., Lo J., Nguyen T.T., Albert J., Abu-Melha A.S., Lin L.M., Gibbs J.L. (2014). Radiographic and Clinical Outcomes of the Treatment of Immature Permanent Teeth by Revascularization or Apexification: A Pilot Retrospective Cohort Study. J. Endod..

[53] Bezgin T., Yilmaz A.D., Celik B.N., Kolsuz M.E., Sonmez H. (2015). Efficacy of Platelet-Rich Plasma as a Scaffold in Regenerative Endodontic Treatment. J. Endod..

[54] Bukhari S., Kohli M.R., Setzer F., Karabucak B. (2016). Outcome of Revascularization Procedure: A Retrospective Case Series. J. Endod..

[55] Song M., Cao Y., Shin S.-J., Shon W.-J., Chugal N., Kim R.H., Kim E., Kang M.K. (2017). Revascularization-Associated Intracanal Calcification: Assessment of Prevalence and Contributing Factors. J. Endod..

[56] Nagy M.M., Tawfik H.E., Hashem A.A.R., Abu-Seida A.M. (2014). Regenerative Potential of Immature Permanent Teeth with Necrotic Pulps after Different Regenerative Protocols. J. Endod..

[57] Nevins A.J., Cymerman J.J. (2015). Revitalization of Open Apex Teeth with Apical Periodontitis using a Collagen-Hydroxyapatite Scaffold. J. Endod..

[58] Silujjai J., Linsuwanont P. (2017). Treatment Outcomes of Apexification or Revascularization in Nonvital Immature Permanent Teeth: A Retrospective Study. J. Endod..

